# Spinal Cord Stimulation Prevents Autonomic Dysreflexia in Individuals with Spinal Cord Injury: A Case Series

**DOI:** 10.3390/jcm12082897

**Published:** 2023-04-16

**Authors:** Soshi Samejima, Claire Shackleton, Raza N. Malik, Kawami Cao, Anibal Bohorquez, Tom E. Nightingale, Rahul Sachdeva, Andrei V. Krassioukov

**Affiliations:** 1 International Collaboration on Repair Discoveries, Faculty of Medicine, University of British Columbia, Vancouver, BC V5Z 1M9, Canada; 2 Division of Physical Medicine and Rehabilitation, Department of Medicine, University of British Columbia, Vancouver, BC V5Z 2G9, Canada; 3 Spinal Cord Program, GF Strong Rehabilitation Centre, Vancouver Coastal Health, Vancouver, BC V5Z 2G9, Canada; 4 School of Sport, Exercise and Rehabilitation Sciences, University of Birmingham, Edgbaston, Birmingham B15 2TT, UK; 5 Centre for Trauma Sciences Research, University of Birmingham, Edgbaston, Birmingham B15 2TT, UK

**Keywords:** spinal cord injury, spinal cord stimulation, autonomic dysreflexia, cardiovascular function, epidural stimulation

## Abstract

Spinal cord injury (SCI) results in severe cardiovascular dysfunction due to the disruption of supraspinal control. Autonomic dysreflexia (AD), an uncontrolled rise in blood pressure in response to peripheral stimuli including common bowel routine, digital anorectal stimulation (DARS), reduces the quality of life, and increases morbidity and mortality. Recently, spinal cord stimulation (SCS) has emerged as a potential intervention to mitigate unstable blood pressure following SCI. The objective of this case series was to test the real-time effect of epidural SCS (eSCS) at the lumbosacral spinal cord, the most common implant location, on mitigating AD in individuals with SCI. We recruited three individuals with cervical and upper thoracic motor-complete SCI who have an implanted epidural stimulator. We demonstrated that eSCS can reduce the elevation in blood pressure and prevent DARS-induced AD. The blood pressure variability analysis indicated that eSCS potentially reduced vascular sympathetic nervous system activity during DARS, compared to without eSCS. This case series provides evidence to support the use of eSCS to prevent AD episodes during routine bowel procedures, improving the quality of life for individuals with SCI and potentially reducing cardiovascular risks.

## 1. Introduction

Spinal cord injury (SCI) results in a disconnect between the supraspinal autonomic control center and the spinal autonomic circuits below the injury [1]. The disrupted autonomic pathways lead to impaired cardiovascular control following SCI, especially with injuries at or above the sixth thoracic spinal cord segment (T6) [2]. Cardiovascular dysfunction not only disturbs activities of daily living and detrimentally impacts the health-related quality of life for individuals with SCI, but also contributes to the deterioration of vascular health, increasing the risk of cerebro- and cardio-vascular diseases [3,4,5,6].

These cardiovascular impairments include episodes of uncontrolled blood pressure (BP) elevation in response to afferent inputs below the injury, a condition known as autonomic dysreflexia (AD) [7,8]. The severity of AD is associated with the completeness of SCI, and 91% of people with cervical SCI present with AD signs [9]. In addition to the loss of supraspinal inhibitory inputs to sympathetic preganglionic neurons (SPNs) below the lesion [10], the neuroplastic changes of SPNs [11,12,13,14,15], changes within propriospinal neurons [16], as well as aberrant plasticity within afferent fibers [17] increase the excitability of the spinal cord in response to peripheral stimulation [18]. AD is commonly caused by bowel routines following SCI, including the regularly used procedure of digital anorectal stimulation (DARS) [19]. DARS can also cause increased sympathetic activity and the development of AD [19]. Inadequate interventions for AD lead to severe cardiovascular conditions, stroke and even death [4,20]. Preventing AD is one of the key health priorities for recovery identified by individuals with cervical and thoracic SCI [21,22]. Current options for managing AD, including non-pharmacological and pharmacological agents [23], frequently have limited effects or significant side effects and a delayed onset of action [24,25]. For instance, some antihypertensive agents (e.g., Nifedipine) can decrease arterial BP below the desired levels, which is sustained for hours, and requires further monitoring and management [24,25]. Alternative options for the management of AD, without significant adverse effects, are needed to improve care for individuals with SCI.

Spinal cord stimulation (SCS) has been used clinically to treat pain since 1967 [26,27]. There is a growing body of evidence indicating that epidural SCS (eSCS), an FDA-approved means to treat pain, potentially modulates spinal circuits via primary afferent inputs, resulting in motor [28,29,30,31] and autonomic [32,33,34,35,36,37,38] recovery following SCI. These studies indicate that the most common positioning of epidural implants is on the lumbosacral spinal cord to target direct innervation of lower extremity muscles and pelvic organs. Furthermore, early work investigating eSCS at the lumbosacral spinal segment demonstrated the long-term effect of stimulation on mitigating AD in four out of five individuals with SCI [39]. However, this study only reported anecdotal evidence (e.g., frequency of AD), without systematic BP measurements, and did not test any real-time effects of eSCS on AD.

Therefore, this study aimed to assess the real-time impact of clinically approved eSCS on preventing DARS-induced AD in three individuals with cervical and upper thoracic motor-complete SCI. We also evaluated the impact of eSCS on vasculature sympathetic nervous system activity during DARS. It was hypothesized that real-time eSCS at the lumbosacral spinal segments could prevent AD during DARS by preventing the increase in sympathetic nervous system activity.

## 2. Methods

This study was approved by the University of British Columbia Clinical Ethics Board (UBC CREB H19-00932) and was conducted in accordance with the Declaration of Helsinki. The participants provided written informed consent prior to their participation.

We recruited individuals with sensorimotor-complete or motor-complete SCI (American Spinal Injury Association impairment scale (AIS) AIS A and B) at T6 or above, who presented with documented AD signs and received the epidural stimulator implantation. We included consecutive participants under the criteria. The participants included two males with traumatic cervical SCI, and one female with traumatic thoracic SCI. All participants underwent implantation of a 16-electrode array (Restore-ADVANCED neurostimulator, Specify 5-6-5, Medtronic, Minneapolis, MN, USA) between T10 and T12 vertebral levels (i.e., the lumbosacral spinal segments) prior to the study. Each individual’s neurological level of injury (NLI) and AIS were determined according to the International Standards for Neurological Classification of Spinal Cord Injury (ISNCSCI) [40].

### 2.1. Study Design and Assessments

**Figure 1:**
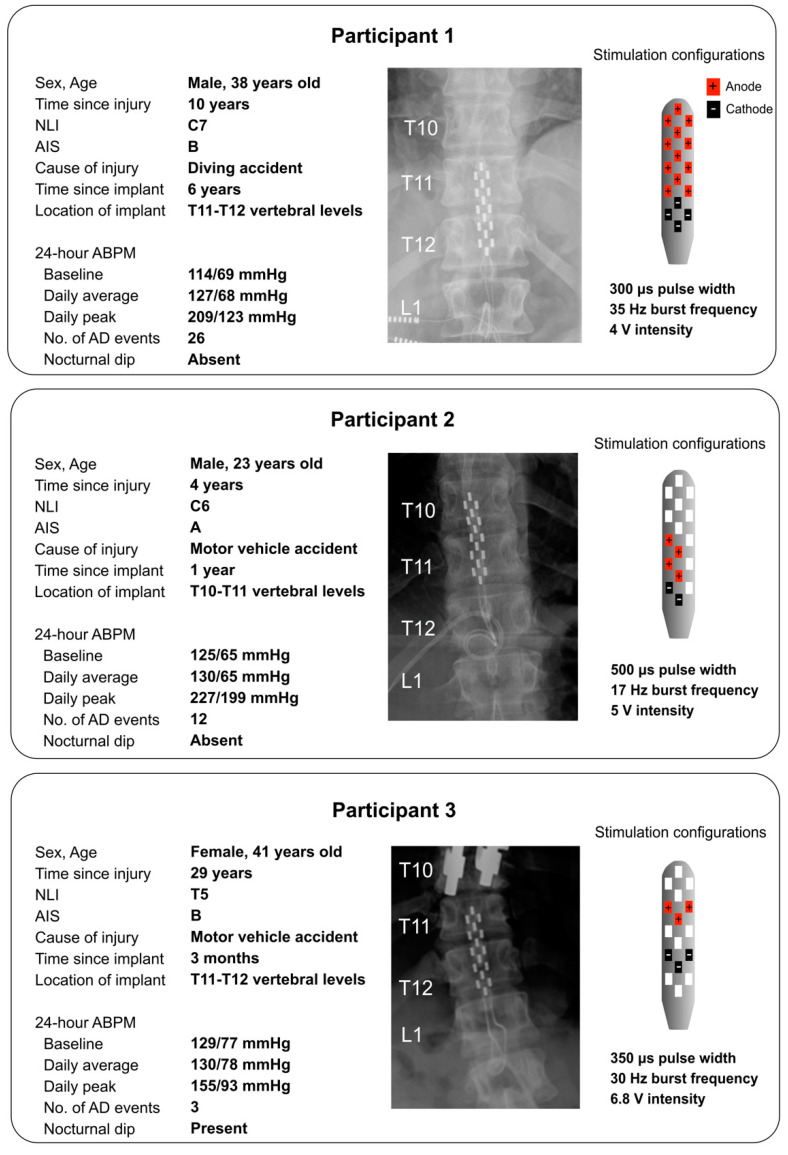
Demographics, blood pressure responses, epidural implant location and stimulation configuration, including active anodic and cathodic electrodes on the implants in three individuals with SCI. Anatomical placement of the 16-electrode array: conventional radiography of the thoracic and lumbar spines displays the position of the 16-electrode array. Stimulation configurations: red identifies anodes and black identifies cathodes. Cathodes were the caudal electrodes in all participants. Abbreviations: ABPM, ambulatory blood pressure monitoring; AD, autonomic dysreflexia; AIS, American Spinal Injury Association Impairment Scale; BP, blood pressure; C, cervical; DBP, diastolic blood pressure; Hz: hertz; SBP, systolic blood pressure; us, microseconds; V, volts.

A summary of participant demographics and injury characteristics are presented in Figure 1. Following screening and informed consent, participants attended their first visit in which their severity of neurological impairment and autonomic cardiovascular dysfunction was assessed using the ISNCSCI exam and 24 h Ambulatory BP Monitoring (ABPM) (Meditech Ltd., Budapest, Hungary) [41], respectively. Prior to the 24 h ABPM, baseline resting BP and heart rate (HR) values were established as the average of three measurements. The ABPM device was applied with appropriate cuff sizes to the non-dominant arm and preprogrammed to record systolic BP (SBP), diastolic BP (DBP) and HR. Cardiovascular parameters were recorded automatically every 15 min during the daytime and every 60 min during the nighttime, based on the individual’s usual sleeping routines.

The second visit involved the DARS procedure with continuous BP monitoring. DARS is a routine procedure to initiate a bowel evacuation and has previously been employed to trigger controlled elevation in arterial BP in individuals with SCI [19,42]. The participants were requested to complete a bowel routine 24 h prior to the assessment. Participants were positioned in the left lateral decubital position and baseline hemodynamics were recorded. Next, an experienced clinician (AVK) delivered DARS in accordance with published recommendations [43]. The index finger was inserted into the rectum and gentle pressure was applied for 30 s. Throughout the assessment, continuous hemodynamic data were recorded to monitor cardiovascular safety and report on the severity of AD experienced by the participants. Beat-by-beat BP and HR were continuously recorded via finger photoplethysmography and five-lead electrocardiography (ECG) using Finapres NOVA (Finapres Medical Systems, Amsterdam, Netherlands), respectively, and brachial BP recorded every minute (Dinamap PRO, GE Healthcare, Chicago, IL, USA). Beat-by-beat BP and HR were sampled at 1000 Hz via an analog-to-digital converter (Powerlab 16/35 System, ADInstruments, Colorado Springs, CO, USA). DARS was performed twice without eSCS and twice with eSCS in a randomized order. Both the clinician and participants remained blinded to the selected eSCS program and BP responses during the DARS procedure. eSCS was selected to target the lumbosacral area involved in bowel control, based on the program in which the cathode electrodes were caudally positioned within the array (Figure 1). To determine the ability of eSCS in preventing an episode of AD, eSCS was initiated 60 s prior to DARS and was sustained for an additional 60 s after DARS was completed.

### 2.2. Data Processing and Analysis

The ABPM data were downloaded for offline analysis using the CardioVisions 1.13.0 software (Meditech Ltd., Budapest, Hungary). Furthermore, participants diarized the time and type of event (e.g., bowel routine initiation) that could have resulted in BP fluctuations and described any potential AD signs and symptoms experienced during the 24 h ABPM period. Based on the clinical definition of AD, in which SBP rises more than 20 mmHg from the baseline resting SBP, all episodes of daily AD were identified for the 24 h period [44].

Offline hemodynamic data analyses of the digitized Finapres signals were performed at a temporal resolution of one millisecond using MATLAB R2021b (Mathworks, Natick, MA, USA). Time series of successive beats were extracted for SBP, DBP and HR. Occasional ectopic beats were corrected by linear interpolation of adjacent normal beats. Baseline BP and HR with and without eSCS were collected for 60 s in the absence of DARS. The magnitude of change in SBP, DBP and HR in response to DARS were calculated as the differences between the average baseline measurements prior to a DARS application and the peak values obtained during DARS. The calculated changes were averaged across two trials per participant under each stimulation condition (eSCS OFF and eSCS ON).

The periodic content of BP variability was assessed using validated wavelet decomposition [45]. This analysis was implemented using the continuous wavelet transform (cwt) function from the MATLAB Wavelet Toolbox, using the default settings with the analytic Morse wavelet. A scalogram was generated to represent the time and frequency domains of BP variability and the amplitude of the frequency power was shown by the intensity of that point using color. By taking the integral of the wavelet power over the selected frequency range, the total power of the scalogram was determined, which is an index of BP variability. The magnitude of the change in lower frequency (LF) power of BP variability was extracted as the difference between the average LF power of the 40 s baseline prior to DARS and the average LF power of the 40 s measurement from the finger insertion. The data period was selected to account for the time from finger insertion (5–10 s) to the completion of DARS (30 s). Specific LF components (i.e., 0.05–0.15 Hz) of BP variability were assessed to estimate vasculature sympathetic activity [46,47]. All data were reported as the mean ± standard deviation.

## 3. Results

**Figure 2:**
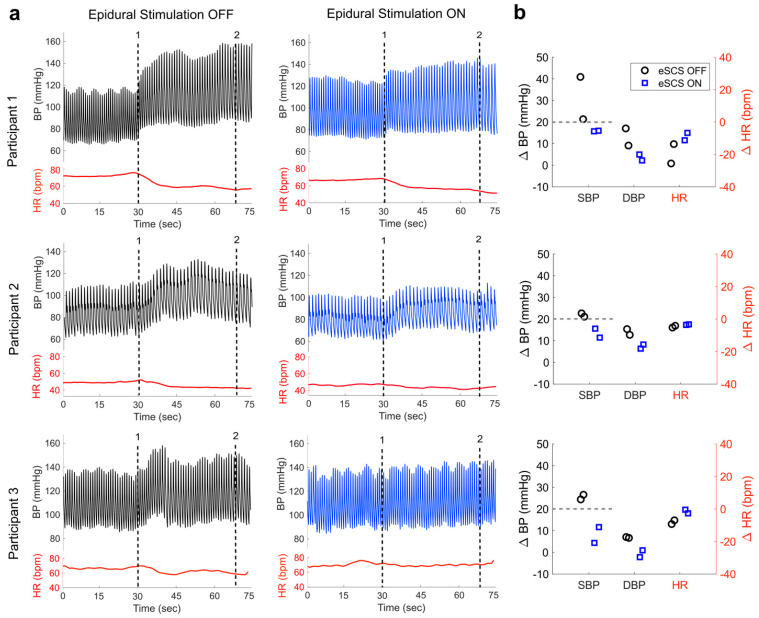

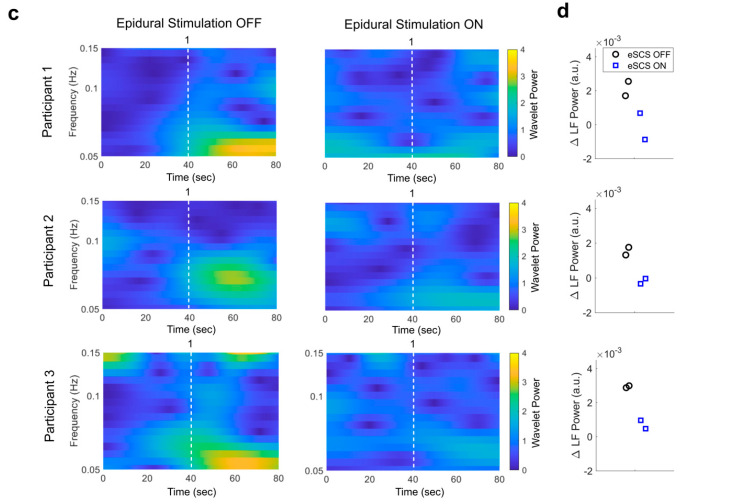
Effect of eSCS on AD during DARS in three individuals with SCI. (**a**) Cardiovascular responses during DARS with eSCS OFF (grey shading) and eSCS ON (blue shading). The first vertical black dotted line (1) shows the start of DARS (i.e., insertion of index finger into the rectum), and the second vertical black dotted line (2) shows the completion of DARS (i.e., removal of finger). (**b**) Changes in SBP, DBP and HR during DARS with (blue symbols) and without (black symbols) eSCS in each participant. During DARS without eSCS, all participants showed an elevation in SBP greater than the 20-mmHg threshold for AD diagnosis (gray dotted lines). However, DARS with eSCS consistently reduced changes in SBP, keeping it below the threshold for AD diagnosis (grey dotted lines). (**c**) Scalogram showing wavelet power (yellow color) at low frequencies (0.05–0.15 Hz, *y*-axis) over time (*x*-axis). Wavelet power scalogram shows increased low frequency power during DARS without eSCS. However, with eSCS, all participants showed a decrease in wavelet power following the start of DARS (white dotted vertical line 1). (**d**) The elevation in low frequency power following DARS was prevented with eSCS ON compared to eSCS OFF in all participants. Abbreviations: a.u., arbitrary unit; BPM, beats per minute; DARS, digital anorectal stimulation; DBP, diastolic blood pressure; eSCS, epidural spinal cord stimulation; HR, heart rate; Hz, hertz; LF, low frequency; mmHg, millimeter of mercury; SBP, systolic blood pressure; ∆, change.

All participants experienced episodes of AD during a daily 24 h period (Figure 1). In addition to the frequent episodes of AD, Participants 1 and 2 had no nocturnal dipping, indicative of severe cardiovascular dysfunction. The effect of eSCS on resting cardiovascular parameters showed that there were minimal changes in resting SBP, DBP and HR between the stimulation conditions in all participants (Table 1). Subsequently, we evaluated the cardiovascular responses to DARS with and without eSCS. DARS without eSCS induced an elevation in SBP of greater than 20 mmHg (Participant 1: change of (Δ)SBP 31 ± 14 mmHg, Participant 2: ΔSBP 22 ± 1 mmHg, Participant 3: ΔSBP 26 ± 2 mmHg) and a simultaneous reduction in HR (Table 1), indicative of AD. Active eSCS during DARS prevented AD, as evidenced by a marginal elevation in SBP of less than the 20 mmHg threshold for AD diagnosis (Participant 1: ΔSBP 16 ± 0.2 mmHg, Participant 2: ΔSBP 13 ± 3 mmHg, Participant 3: ΔSBP 8 ± 5 mmHg) and a minimal reduction in HR (Table 1, Figure 2a,b).

Wavelet decomposition analysis of BP variability, in the absence of eSCS, showed a higher LF wavelet power concomitant with the elevation of SBP during DARS (Table 1). However, real-time eSCS resulted in decreased LF wavelet power (Participant 1: 0.0021 ± 0.0006 vs. −0.0001 ± 0.0011, Participant 2: 0.0015 ± 0.0003 vs. −0.0002 ± 0.0002, Participant 2: 0.0029 ± 0.0001 vs. −0.0007 ± 0.0003), concomitant with the mitigation of AD during DARS (Figure 2c,d).

## 4. Discussion

Severe AD symptoms during bowel management (e.g., DARS) are associated with a lower quality of life following SCI [48,49,50]. In addition, the labile BP is the leading cause of cardiovascular morbidity and mortality after SCI [6,51,52,53]. Despite the risk of potentially life-threatening episodes of AD associated with DARS, it is still a commonly used method for bowel evacuation following SCI. In this case series, we demonstrated the effect of real-time eSCS on preventing AD during DARS in three individuals with cervical and upper thoracic motor-complete SCI. Our results show that real-time eSCS during DARS decreased the elevation of LF wavelet power in BP variability, indicating reduced vasculature sympathetic nerve activity [46,47]. Therefore, clinically available lumbosacral eSCS is potentially a fast-acting and effective alternative to pharmacological management of AD, which could reduce cardiovascular risk and improve the health-related quality of life.

Previous neurostimulation studies have shown that by targeting the SPNs directly, SCS can mitigate AD [42]. In this study, we used eSCS programs where the active electrodes (i.e., cathode) stimulated the caudal lumbosacral spinal segments. Therefore, we show that eSCS delivered at the lumbosacral region (i.e., outside the T1–L2 segments that contain SPNs) can similarly effectively mitigate AD. These results suggest that SCS over the caudal lumbosacral spinal segments can impact the SPNs potentially via propriospinal neurons [32]. This contrasts previous studies which proposed eSCS at the lower thoracic spinal segments as a “hemodynamic hot spot” for BP control [33,54]. Thus, it is possible that eSCS at the lumbosacral spinal segments, which targets motor and pelvic organ function, could be repurposed for the recovery of cardiovascular control following SCI.

**Figure 3:**
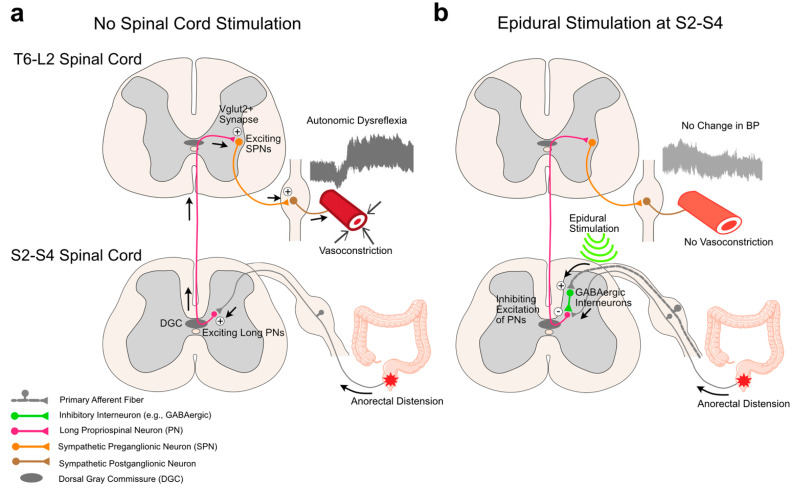
Potential mechanism of epidural spinal cord stimulation for mitigating autonomic dysreflexia. This figure shows the effect of eSCS at the caudal lumbosacral spinal segments (S2–S4) on visceral stimuli in the intact and injured spinal cord, at or above T6. (**a**) Without eSCS, visceral inputs by anorectal distention activate excitatory interneurons (red) through afferent fibers. The excitatory inputs polysynaptically ascend to thoracolumbar spinal segments (T6–L2), likely via long propriospinal neurons in the dorsal gray commissure (DGC) [16]. The ascending inputs activate sympathetic preganglionic neurons (SPNs, orange) via glutamatergic synapses (e.g., vesicular–glutamate transporter positive (Vglut2+) synapses), increasing vasculature of the sympathetic nervous system activity, leading to autonomic dysreflexia [13,14]. (**b**) With eSCS, primary afferent inputs (gray with myelination) potentially activate inhibitory interneurons (e.g., GABAergic interneurons, green) [56,57]. The inhibitory interneurons may inhibit the activation of excitatory interneurons (red), which results in no excitation of SPNs to prevent vasoconstriction related to visceral inputs from S2 to S4.

The most plausible mechanism for the effect of SCS on AD relies on the gate control theory [55]. This theory proposes that primary afferent inputs induce inhibitory mechanisms to close the gate to visceral or noxious inputs at the spinal cord level. Based on the gate control theory and the location of the active electrodes in this study, we propose that eSCS-induced primary afferent inputs at the sacral spinal segments likely inhibited the activation of excitatory interneurons (e.g., long propriospinal neurons) [16] in response to visceral inputs (i.e., DARS) via inhibitory interneurons [32]. These inhibitory interneurons (e.g., GABAergic interneurons) can be activated via large afferent fibers [56,57]. A previous study showed that DARS increased the vasculature sympathetic outflow, measured by norepinephrine, in individuals with SCI [19]. In our study, the smaller changes in LF wavelet power indicate that eSCS potentially inhibited the excitation of the vascular sympathetic nervous system activity in all participants. Based on our case series results, we hypothesize that eSCS prevented AD through inhibitory interneurons in the sacral spinal cord, potentially preventing the activation of maladapted sympathetic spinal circuits controlling hyperexcitable SPNs in T6–L2 spinal segments (Figure 3).

There are several limitations to this study. First, we tested the effect of eSCS in only three participants. Consequently, investigations in a larger cohort, with more diverse injury and demographic characteristics are warranted to confirm the efficacy of eSCS for preventing AD in this population. Second, this study presents an experimental AD assessment in participants under a controlled laboratory manner to ensure cardiovascular safety. The preliminary findings of this study need to be translated into investigating the effect of eSCS on uncontrolled hypertensive episodes across various daily activities (e.g., bladder distension, catheterization, and sexual activity) or iatrogenic clinical procedures (e.g., urodynamics, penile vibrostimulation). Future studies also need to examine the long-term effect of eSCS on targeting the sacral spinal segments for mitigating AD. Third, although eSCS is approved by the FDA for pain management, the implantation of eSCS involve several risks, including surgical complications, cost and pulse generator battery life. The community needs to test whether a non-invasive means, such as tSCS which has not been approved by the FDA, can be an alternative therapy for mitigating AD [42]. Finally, this clinical study should be reverse-translated into rat SCI models to dissect activated and inhibited spinal neurons during the intervention, to better understand the precise mechanisms of action.

## 5. Conclusions

Lumbosacral eSCS prevented AD induced by DARS and decreased LF wavelet power in individuals with cervical and upper thoracic motor-complete SCI. More evidence is needed to clarify the underlying inhibitory mechanisms of eSCS and verify the best location of spinal cord stimulation to optimize the prevention of AD. eSCS could serve as a fast-acting therapeutic tool for mitigating uncontrolled BP fluctuations following SCI, leading to a decreased risk of associated cardiovascular consequences and an improved quality of life for individuals with SCI.

## Figures and Tables

**Table 1:** Cardiovascular responses at rest (supine) and in response to DARS, with and without eSCS in individuals with SCI.

	Participant 1		Participant 2		Participant 3	
	Without eSCS	With eSCS	Without eSCS	With eSCS	Without eSCS	With eSCS
Baseline SBP (mmHg)	110 ± 10	118 ± 12	102 ± 2	96 ± 1	134 ± 1	137 ± 4
Baseline DBP (mmHg)	63 ± 7	66 ± 10	62 ± 3	53 ± 2	93 ± 5	91 ± 1
Baseline HR (bpm)	71 ± 8	71 ± 14	47 ± 1	46 ± 1	69 ± 0.1	70 ± 1
Baseline LF wavelet power (a.u.)	0.0047 ± 0.0030	0.0051 ± 0.0015	0.0050 ± 0.0044	0.0050 ± 0.0013	0.0058 ± 0.0014	0.0052 ± 0.0003
∆ SBP during DARS (mmHg)	31 ± 14	16 ± 0.2	22 ± 1	13 ± 3	26 ± 2	8 ± 5
∆ DBP during DARS (mmHg)	13 ± 6	4 ± 2	14 ± 2	7 ± 1	7 ± 0.2	−1 ± 2
∆ HR during DARS (bpm)	−20 ± 8	−9 ± 3	−5 ± 1	−3 ± 0.2	−8 ± 2	−2 ± 2
∆ LF wavelet power during DARS (a.u.)	0.0021 ± 0.0006	−0.0001 ± 0.0011	0.0015 ± 0.0003	−0.0002 ± 0.0002	0.0029 ± 0.0001	0.0007 ± 0.0003

Abbreviations: a.u., arbitrary unit; bpm, beats per minute; DARS, digital anorectal stimulation; DBP, diastolic blood pressure; eSCS, epidural spinal cord stimulation; HR, heart rate; LF, low frequency; mmHg, millimeter of mercury; SBP, systolic blood pressure; SCI, spinal cord injury; ∆, change. Data are represented as the mean of two measurements ± standard deviation.

## Data Availability

The data that support the findings of this study can be available contacting the corresponding author via e-mail.
